# Implementation of a Novel Faculty-Resident Education Subcommittee Model in a Radiation Oncology Residency Program: A Technical Report

**DOI:** 10.7759/cureus.117163

**Published:** 2026-09-29

**Authors:** Brady S Laughlin, Kashif Majeed, Sean T McCauley, Randa Tao, Jonathan Ashman, Terence Sio, William Rule, Sarah James, Joshua Niska, Safia Ahmed, Nathan Y Yu, Fan Yang, Jean-Claude M Rwigema, Lisa A McGee

**Affiliations:** 1 Radiation Oncology, Mayo Clinic in Arizona, Phoenix, USA; 2 Radiation Oncology, Mayo Clinic in Arizona, Scottsdale, USA; 3 Radiation Oncology, Santa Clara Valley Medical Center, San Jose, USA

**Keywords:** curriculum development and evaluation, educational innovation, faculty engagement, graduate medical education (gme), radiation oncology education, residency training, resident engagement

## Abstract

At our institution, a small program leadership team oversees radiation oncology residency education. We implemented a faculty-resident subcommittee model to broaden participation and organize program-development activities. During the 2024-2025 academic year, five subcommittees were established to address recruitment, medical student engagement, program growth and optimization, curriculum, and education innovation and learning environment optimization. Each subcommittee was co-chaired by the program director and education program coordinator and included two to three additional faculty members. Four residents, representing postgraduate years (PGYs) 2-5, each participated in at least one subcommittee. Participation and implementation outputs were summarized descriptively. Twelve of 15 faculty members and all 4 residents participated. Outputs included two virtual recruitment events, four faculty members' participation in application review, on-site and virtual medical student open houses, development of an advanced radiation medicine fellowship, rotation and curriculum revisions, nuclear medicine workflows, centralized educational resources, and stakeholder surveys. The structure engaged faculty and residents in defined program-development activities and generated initiatives across five educational domains. This single-program experience suggests that the model was feasible. Longitudinal evaluation of the impact of this model is needed.

## Introduction

The Accreditation Council for Graduate Medical Education (ACGME) assigns the program director (PD) substantial responsibility for program administration, resident education and evaluation, faculty oversight, and regulatory compliance. A program coordinator is also required, and additional leadership positions, such as an assistant program director (APD), may be established based on program needs. In our program, the leadership team comprises the PD, APD, and education program coordinator (EPC). ACGME also requires faculty participation in the Clinical Competency Committee (CCC) and Program Evaluation Committee (PEC). The CCC reviews resident evaluations and Milestones at least semiannually, whereas the PEC conducts the Annual Program Evaluation and guides continuing program improvement [1,2]. Broader faculty involvement beyond these designated roles is not required by the ACGME, and the formal inclusion of additional faculty within the residency education structure is not customary.

Historically, our department had a single Education Committee led by the PD, APD, and EPC, with an additional 8-12 faculty members participating from a total of 15 physician faculty. The residency program consisted of four residents, one per postgraduate year (PGY). Resident physicians were not historically included as members of the Education Committee.

Radiation oncology residency education presents distinct programmatic challenges given the small size of many training programs, the breadth of disease-site-specific knowledge, technical and procedural competencies, multidisciplinary clinical experiences, and the need to adapt training to rapidly evolving technologies, treatment paradigms, and graduation requirements. Within this context, our larger Education Committee worked collaboratively to ensure high-quality resident education, provide program oversight, maintain compliance, and continually evolve the program in response to the changing needs of both the residency program and individual residents. Program leadership observed, however, inconsistent faculty engagement and limited ownership of initiatives within this large-group structure. Prior publications describe residency redesign, PEC reform, and multiprogram advisory structures, but the literature identified for this report did not describe a subcommittee faculty-resident model [3-5]. Literature on group size, participation, social loafing, and groupthink suggests that large committees can experience coordination and participation challenges, and supports smaller committees to promote meaningful participation and effective decision-making [6-10].

Following a transition in program leadership prior to the 2024-2025 academic year (AY), five priority areas for program evolution and improvement were identified based on feedback from both resident physicians and faculty. In response, five subcommittees were established, focused on recruitment, medical student engagement, program growth and optimization, curriculum, and education innovation and learning environment optimization. Each subcommittee functioned as a task force responsible for developing and implementing changes and new initiatives within its area of focus and reporting its work to the Education Committee. This structure was intended to distribute educational leadership and responsibility across faculty and residents while addressing priorities particularly relevant to a small radiation oncology training program. This report describes implementation of the subcommittee model and summarizes first-year programmatic outputs during the 2024-2025 AY.

## Technical report

Program setting and implementation

This descriptive implementation report was conducted within a single radiation oncology residency program during the 2024-2025 AY. The department included 15 physician faculty members and 4 radiation oncology residents, with 1 resident at each level from PGY-2 through PGY-5. The PGY-1 internship was completed separately as a transitional year program at our institution before entry into radiation oncology training as a PGY-2. At the beginning of the AY, 12 of 15 faculty members expressed interest in membership on the Education Committee. Following a transition in program leadership, the committee was reorganized into five subcommittees: recruitment, medical student engagement, program growth and optimization, curriculum, and education innovation and learning environment optimization. The PD and EPC co-chaired each subcommittee, which included 2-3 additional faculty members. The 10 participating faculty members other than the PD and EPC were assigned to at least one subcommittee, and each of the four residents joined at least one subcommittee. The organizational structure is shown in Figure 1. The subcommittees met regularly and incorporated feedback and ideas from both faculty and residents.

**Figure 1:**
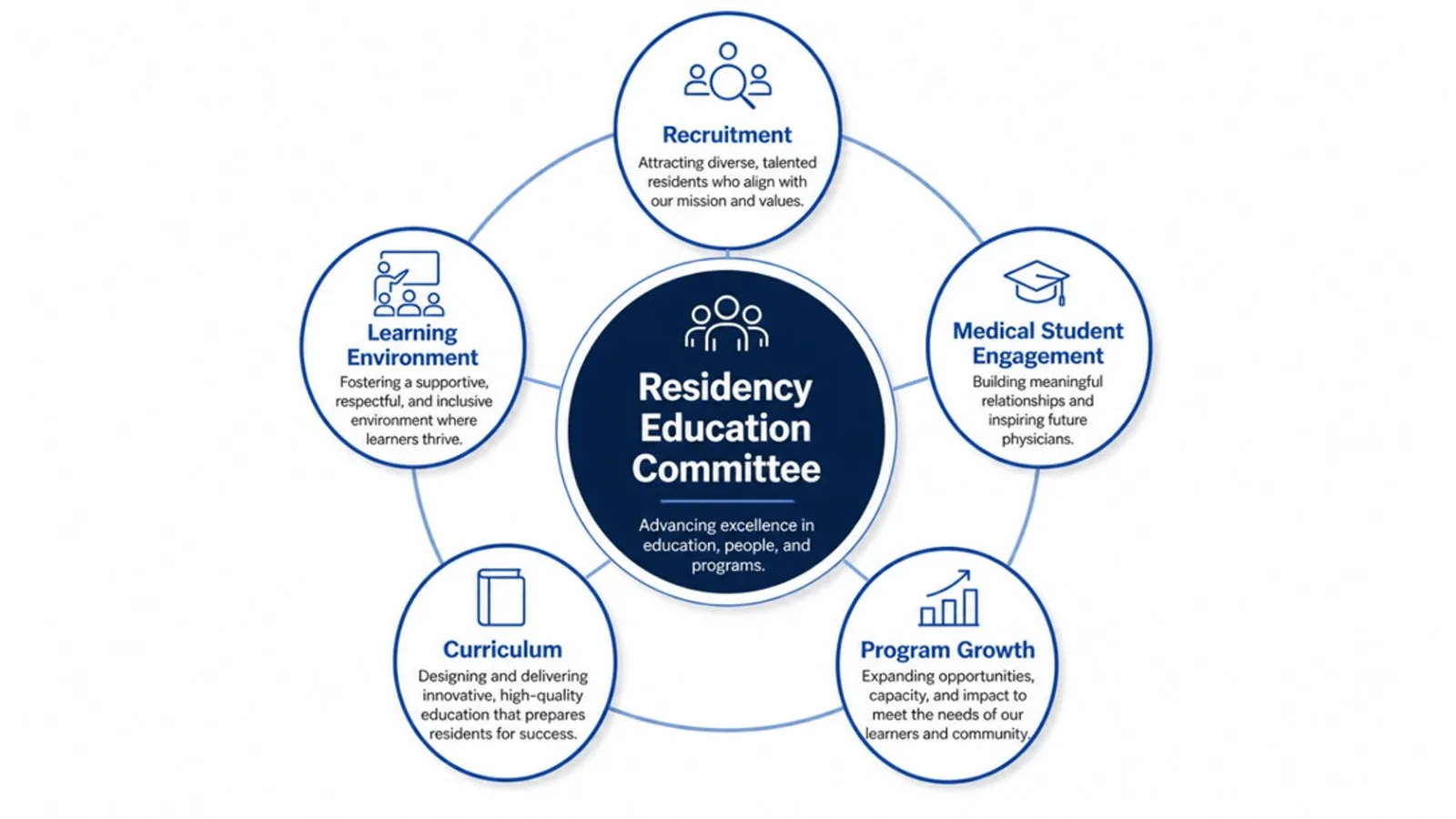
Five-pillar education subcommittee model within the radiation oncology residency program. Each pillar represents a distinct subcommittee: recruitment, medical student engagement, program growth and optimization, curriculum, and learning environment. Each includes the program director, education program coordinator, faculty, and resident representatives to foster distributed leadership and increase faculty involvement in program development.

Implementation outcomes

All 12 faculty members of the Education Committee and all 4 resident physicians participated in at least one subcommittee. Outside the PD and EPC, three faculty members served on more than one committee. Each subcommittee was assigned a distinct primary area of focus, as summarized in Table 1.

**Table 1:** Subcommittee structure with a focus on early outcomes during the 2024-2025 academic year in a radiation oncology residency program. The table outlines each subcommittee’s main domain: recruitment, student engagement, program growth, curriculum, and learning environment optimization. It highlights initiatives and outcomes demonstrating this educational leadership model's impact.

Subcommittee	Primary focus	Outcomes
Recruitment	Applicant pipeline and visibility	Virtual applicant events, expanded faculty application review, streamlined interview day
Medical student engagement	Early exposure and relationship-building	Open houses, lectures, social media outreach
Program growth and optimization	Strategic expansion and infrastructure	Development of advanced fellowship, improved orientation structure
Curriculum	Clinical and didactic modernization	Rotation redesign, board prep integration, biostatistics curriculum, nuclear medicine AU pathway
Learning environment optimization	Culture, organization, feedback systems	Centralized resource library, standardized conference scheduling, climate surveys, structured mentorship touchpoints

Recruitment

The Recruitment Subcommittee developed a longitudinal outreach strategy to increase the program’s visibility and strengthen applicant engagement. Activities included participation in national and regional specialty events and medical student interest-group programs. The subcommittee hosted two virtual recruitment events, engaged four faculty members in residency application review, and reduced the number of interviews scheduled per day in response to resident and applicant feedback.

Medical student engagement

The Medical Student Engagement Subcommittee developed a longitudinal strategy to provide medical students with early exposure to radiation oncology [11,12]. Activities included participation in specialty interest groups, delivery of lectures and panel discussions, involvement in career-exploration events, and on-site and virtual departmental open houses [13]. Social media content was used to promote the residency program and maintain engagement with prospective applicants. These activities were intended to increase awareness of radiation oncology and support longitudinal faculty-student connections and mentorship [14].

Program growth and optimization

The Program Growth and Optimization Subcommittee collaborated with departmental leadership to develop and secure institutional approval for an advanced radiation medicine fellowship. The first fellow was recruited during the following 2025-2026 AY, and successfully joined the department for the 2026-27 AY. The fellowship was promoted via a dedicated institutional website. Subcommittee members were also responsible for interviewing fellowship candidates.

Curriculum

Curriculum changes were undertaken to align resident education with board requirements, career goals, and clinical practice needs. Board-preparation sessions were added, and existing sessions were reviewed for clinical relevance. Faculty teaching assignments were redistributed to improve scheduling flexibility and reduce concentrated teaching burden for individual faculty. Rotations were reorganized to provide dedicated board-preparation time and greater scheduling flexibility. Historically, radiation oncology residents completed a mandatory two-week pediatric medical oncology rotation during their pediatric radiation oncology block, limiting the time available for radiation oncology training in this complex and challenging disease site. To better support achievement of radiation oncology case requirements and development of the knowledge and experience necessary for independent practice and board preparation, the pediatric medical oncology rotation was changed from a required rotation to an elective option during the final year of training. This change provided residents with an additional two weeks of dedicated pediatric radiation oncology training.

Disease-site faculty education leads were appointed to guide structured educational sessions. A comprehensive biostatistics lecture series was integrated into the core curriculum, and a radiobiology self-study library was created. Planning began to incorporate dosimetry into the physics curriculum. A partnership with Nuclear Medicine was established to provide relevant clinical exposure and support completion of experiences related to Authorized User eligibility. The subcommittee also revised resident orientation by removing low-value activities and adjusting the schedule to enable earlier clinical integration.

Education innovation and learning environment optimization

The Education Innovation and Learning Environment Optimization (EILEO) subcommittee focused on improving the organization and accessibility of educational resources. The subcommittee established a centralized library containing recorded lectures and shared slide decks, consolidated previously fragmented SharePoint education pages, standardized morning-conference scheduling, and eliminated redundant calendars. These changes standardized access to educational materials, schedules, and program communications. The committee did not formally measure the effect on learning-environment quality during the reporting period.

The subcommittee distributed anonymous surveys to faculty, current residents, and alumni, and used the findings to inform goals for the subsequent AY. Structured resident-program leadership meetings were incorporated into the calendar twice monthly, along with quarterly one-on-one mentor lunches.

Subcommittee initiatives and metrics are summarized in Table 2.

**Table 2:** Subcommittee initiative and metric summary. AY: academic year, EILEO: Education Innovation and Learning Environment Optimization.

Subcommittee	Initiative	Implementation timeframe	Observable output/metric
Recruitment	Virtual recruitment events	July 2024-March 2025	2 events
	Expanded application review	July 2024-March 2026	4 faculty reviewers
	Interview-day redesign	July 2024-March 2027	Fewer interviews scheduled per day
Medical student engagement	Open houses	2024-2025 AY	Virtual and in-person events
	Medical student outreach	2024-2025 AY	Social media, lectures, specialty-interest activities
Program growth and optimization	Advanced radiation medicine fellowship	September 2024-February 2025	Fellowship developed/approved; first fellow recruited for 2026-27 AY
Curriculum	Resident orientation redesign	July 2024	Low-value activities removed; earlier clinical integration
	Rotation redesign	2024-2025 AY	Pediatric medical oncology changed from required rotation to elective
	Board preparation	2024-2025 AY	Dedicated board-preparation sessions/time added
	Biostatistics/radiobiology	2024-2025 AY	Lecture series and self-study library established
	Nuclear medicine education for authorized user eligibility	2024-2025 AY	Clinical workflow established for experiences relevant to Authorized User eligibility
EILEO	Educational resource consolidation	2024-2025 AY	Centralized lecture/slide library and consolidated SharePoint resources
	Scheduling infrastructure	2024-2025 AY	Standardized conference scheduling; redundant calendars eliminated
	Stakeholder feedback	2024-2025 AY	Faculty, resident, and alumni surveys deployed
	Resident-leadership communication	2024-2025 AY	Twice-monthly meetings
	Mentorship	2024-2025 AY	Quarterly mentor lunches

## Discussion

This report describes the implementation of a five-subcommittee structure spanning recruitment, medical student engagement, program growth, curriculum, and learning environment optimization. Related literature addresses residency redesign, PEC reform, and multiprogram advisory structures, but these publications describe alternate governance models [3-5]. The present model reorganized educational work into defined domains while retaining oversight by the PD and EPC. This approach is relevant because ACGME requirements assign substantial administrative, educational, assessment, and compliance responsibilities to program leadership [1,2]. Burnout and distress have also been documented in a national survey of internal medicine program directors, supporting attention to leadership workload and institutional support [15].

Committees can support program evaluation and change within graduate medical education [4,5]. ACGME has required PECs since 2014 [4,16]. A redesigned PEC has been reported to generate actionable programmatic feedback [4]. The pre-existing Education Committee within our department was not optimally structured and was too large to drive widespread change quickly. Classic group research suggests that working groups of 3-8 members can be more productive than larger groups, although optimal size depends on the task and context [6,7]. Smaller committees may support more efficient decision-making by easing scheduling, shortening discussions, and facilitating consensus [17,18]. Smaller groups may also reduce social loafing, in which individual effort and ownership can decline as group size increases [8]. Larger groups can reduce opportunities for individual participation and can experience process losses or coordination inefficiencies [6,9]. Groupthink can occur when pressures for agreement suppress dissent and critical evaluation; however, it is a group decision-making risk rather than an inevitable consequence of group size alone [10]. These findings supported dividing our large Education Committee into smaller subcommittees designed as action-oriented bodies, each focused on a defined residency program improvement initiative.

During one AY, the subcommittee structure generated initiatives across five educational domains. Observable outputs included fellowship development, rotation and curriculum changes, centralized educational resources, structured stakeholder feedback, residency recruitment events, and expanded medical student outreach. Medical student engagement and recruitment activities included 21 social media posts, virtual and in-person open houses, three lectures at local medical schools, and research mentorship for four medical students [11,13]. The use of social media, the in-person open house, and targeted lectures at local medical schools represented new initiatives developed through the Medical Student Engagement Subcommittee. Research mentorship also expanded, largely as a result of connections established through the in-person open house. These activities demonstrate implementation activity but do not establish improvements in educational quality, learner performance, faculty workload, or the learning environment. Mentorship and sustained faculty engagement nevertheless remain important components of learner support [14].

Faculty empowerment may represent an important mechanism through which the subcommittee structure supports educational innovation. Empowerment can foster creativity, professional judgment, and faculty ownership of educational activities [19]. The present structure created opportunities for collaboration and participation in program development; however, faculty empowerment and leadership development were not measured directly.

The initiatives address several current challenges in radiation oncology training. Rotation optimization, centralized educational resources, and development of nuclear medicine workflows support current training requirements and experiences relevant to Authorized User eligibility [2,20]. Recruitment and medical student engagement activities align with ongoing radiation oncology workforce-development concerns [21]. Curriculum and learning-environment activities are also consistent with contemporary competency-based radiation oncology training frameworks [22,23].

It is uncertain how adaptable our subcommittee model may be to other radiation oncology residency programs and specialties. Multicenter implementation of the Introductory Radiation Oncology Curriculum across 55 programs demonstrates that structured educational interventions can be disseminated across institutions [24-28]. However, the present subcommittee model is a different type of structured initiative than a curriculum, and has been implemented in only one program. Broader-scale feasibility and effects in other settings remain unknown.

This report has several limitations. It describes implementation within a single radiation oncology residency program over one AY and does not include a comparator or standardized pre-implementation measures of faculty or resident engagement. The reported outcomes primarily represent participation counts and programmatic outputs rather than validated measures of resident learning, faculty workload, burnout, recruitment outcomes, or learning-environment quality. Faculty participation may have been influenced by self-selection and other unexplored confounders. Consequently, the findings demonstrate implementation feasibility and early programmatic activity but do not establish effectiveness or generalizability. Sustainability will require continued institutional support, protected time, role clarity, and integration of faculty development with recognition and advancement structures. Lastly, we do not have formal survey data from the faculty describing perspectives of this change.

## Conclusions

Implementation of a five-subcommittee educational structure was feasible within one radiation oncology residency program and engaged 12 of 15 faculty members and all 4 residents in program-development activities. The structure organized faculty and resident contributions across five defined educational domains and generated multiple program-level initiatives during one AY. Longer-term evaluation is needed to determine its effects on faculty workload, resident educational outcomes, recruitment, and the learning environment.
